# Automatic Radiotherapy Planning for Glioblastoma Radiotherapy With Sparing of the Hippocampus and nTMS-Defined Motor Cortex

**DOI:** 10.3389/fneur.2021.787140

**Published:** 2022-01-14

**Authors:** Michaela Schuermann, Yvonne Dzierma, Frank Nuesken, Joachim Oertel, Christian Rübe, Patrick Melchior

**Affiliations:** ^1^Department of Radiotherapy and Radiation Oncology, Saarland University Hospital, Homburg, Germany; ^2^Faculty of Medicine, Saarland University, Saarbrücken, Germany; ^3^Department of Neurosurgery, Saarland University Hospital, Homburg, Germany

**Keywords:** navigated transcranial magnetic stimulation (nTMS), glioblastoma, hippocampus, Auto-Planning, radiotherapy

## Abstract

**Background:**

Navigated transcranial magnetic stimulation (nTMS) of the motor cortex has been successfully implemented into radiotherapy planning by a number of studies. Furthermore, the hippocampus has been identified as a radiation-sensitive structure meriting particular sparing in radiotherapy. This study assesses the joint protection of these two eloquent brain regions for the treatment of glioblastoma (GBM), with particular emphasis on the use of automatic planning.

**Patients and Methods:**

Patients with motor-eloquent brain glioblastoma who underwent surgical resection after nTMS mapping of the motor cortex and adjuvant radiotherapy were retrospectively evaluated. The radiotherapy treatment plans were retrieved, and the nTMS-defined motor cortex and hippocampus contours were added. Four additional treatment plans were created for each patient: two manual plans aimed to reduce the dose to the motor cortex and hippocampus by manual inverse planning. The second pair of re-optimized plans was created by the Auto-Planning algorithm. The optimized plans were compared with the “Original” plan regarding plan quality, planning target volume (PTV) coverage, and sparing of organs at risk (OAR).

**Results:**

A total of 50 plans were analyzed. All plans were clinically acceptable with no differences in the PTV coverage and plan quality metrics. The OARs were preserved in all plans; however, overall the sparing was significantly improved by Auto-Planning. Motor cortex protection was feasible and significant, amounting to a reduction in the mean dose by >6 Gy. The dose to the motor cortex outside the PTV was reduced by >12 Gy (mean dose) and >5 Gy (maximum dose). The hippocampi were significantly improved (reduction in mean dose: ipsilateral >6 Gy, contralateral >4.6 Gy; reduction in maximum dose: ipsilateral >5 Gy, contralateral >5 Gy). While the dose reduction using Auto-Planning was generally better than by manual optimization, the radiated total monitor units were significantly increased.

**Conclusion:**

Considerable dose sparing of the nTMS-motor cortex and hippocampus could be achieved with no disadvantages in plan quality. Auto-Planning could further contribute to better protection of OAR. Whether the improved dosimetric protection of functional areas can translate into improved quality of life and motor or cognitive performance of the patients can only be decided by future studies.

## Introduction

While it has long been observed that brain radiotherapy can cause neurocognitive sequelae (1–5), only recently have technological advances, such as image-guided radiotherapy, intensity- and volumetric-modulated therapy, and improved treatment planning algorithms, i.e., automatic inverse optimization, allowed selective protection of critical brain structures. Hence, increasing focus is being placed on identifying structures vulnerable to radiation-induced deficits and protecting them by selective dose-shaping.

Most research in this area has focused on the hippocampus, whose dentate gyrus presents a neuronal progenitor cell niche in the adult brain, thus meriting particular dose sparing in radiotherapy (6). A higher dose to the hippocampus has been observed to correlate with neurocognitive deficits, particularly regarding verbal memory and higher cognitive functions (7–13). Possibly related to cognitive effects, a dose-dependent volume loss has been observed for a variety of cortical and sub-cortical regions (14–17). Nagtegaal et al. (17) reported 0.16% hippocampus volume loss per year and per Gray mean dose, translating this into an increase in hippocampal age (derived by a nomogram-based method) of between 2 and 20 years for doses up to 50 Gy.

Radiation-induced cortical atrophy of motor-eloquent regions, such as the precentral cortex, has furthermore been linked with impaired fine motor skills (18). In addition to motor deficits observed after stereotactic radiosurgery (19), dose to the precentral gyrus has been correlated with impaired verbal and working memory, attention, and executive functions (12).

Consequently, a number of studies have included the motor cortex as a radiosensitive organ in radiotherapy treatment planning (20–26). All these studies have employed navigated transcranial magnetic stimulation (nTMS) as a reliable technique to non-invasively define the motor cortex, which has proven useful in pre-surgical mapping (27–36). Most studies concentrated on the stereotactic treatment of metastases or arterio-venous malformations (AMV's); however, radiotherapy treatment planning of gliomas can be expected to offer even more optimization potential due to larger target volumes and highly modulated treatment plans as compared with stereotactic treatment. To our knowledge, only one study so far considered adjuvant radiotherapy for high-grade gliomas (21). Re-optimizing volumetric-modulated arc therapy (VMAT) treatment plans for 30 patients with an additional optimization objective on the nTMS-defined motor area, Diehl et al. could achieve a significant reduction of dose to the motor area by 14.3%.

The aim of our study is to further investigate the possibilities of motor cortex sparing in the treatment of high-grade gliomas. Beyond sparing the nTMS-defined motor cortex, we emphasize the importance of maintaining hippocampus sparing. In a previous study for brain metastases, we could show that the inclusion of motor cortex objectives in treatment planning without additional hippocampus objectives may even increase hippocampus dose (26). Hence, we here assess the possibility of the combined motor cortex and hippocampus optimization in radiotherapy treatment of glioblastoma (GBM).

Past years have witnessed an increasing use of automated planning algorithms to improve dose optimization, which have been shown to contribute to improved sparing of organs at risk (OAR) with no accompanying loss in target coverage (37–41). Therefore, in the present study, the manual re-optimization is compared with the results from an automated planning engine to assess whether further improvements in this highly complex scenario are achievable.

## Patients and Methods

In this retrospective study, patients with motor-eloquent GBMs who underwent nTMS-based surgical resection and radiotherapy between 2013 and 2018 were replanned and analyzed.

### nTMS Mapping and Preparation in the Radiotherapy Treatment Planning System (TPS)

The nTMS of the patients was performed on a pre-operative MRI with contrast-enhanced T1-weighed Magnetization Prepared - RApid Gradient Echo (MP-RAGE) in the axial direction on a 1.5 or 3 T scanner (Magnetom Symphony-TIM 1.5 T, Magnetom Skyra 3.0 T, Siemens, Erlangen, Germany). The nTMS motor mapping was done using the Nexstim navigated brain stimulation (NBS) system 4.3 according to the protocol by Picht et al. (28). The patient was seated in a reclined position, and surface electromyography electrodes were attached to the abductor pollicis brevis, first dorsal interosseous, abductor digiti minimi, anterior tibial, and/or plantar muscles. The resting motor threshold (RMT) was identified by stimulating the presumed localization of the hand knob with different coil locations and orientations. The lowest nTMS stimulus intensity in which a 50 μV MEP response (peak-to-peak amplitude) is elicited in five out of 10 stimulations is generally defined as the RMT. The subsequent mapping was then carried out using 110–130% RMT, varying the coil position over the tumor and adjacent gyri. Positive responses were defined as MEP amplitudes above 50 μV and were marked as motor-eloquent stimulation on the MRI. The resulting collection of points is generally taken to represent the location of the primary motor cortex of the hand and lower extremity.

**Figure 1:**
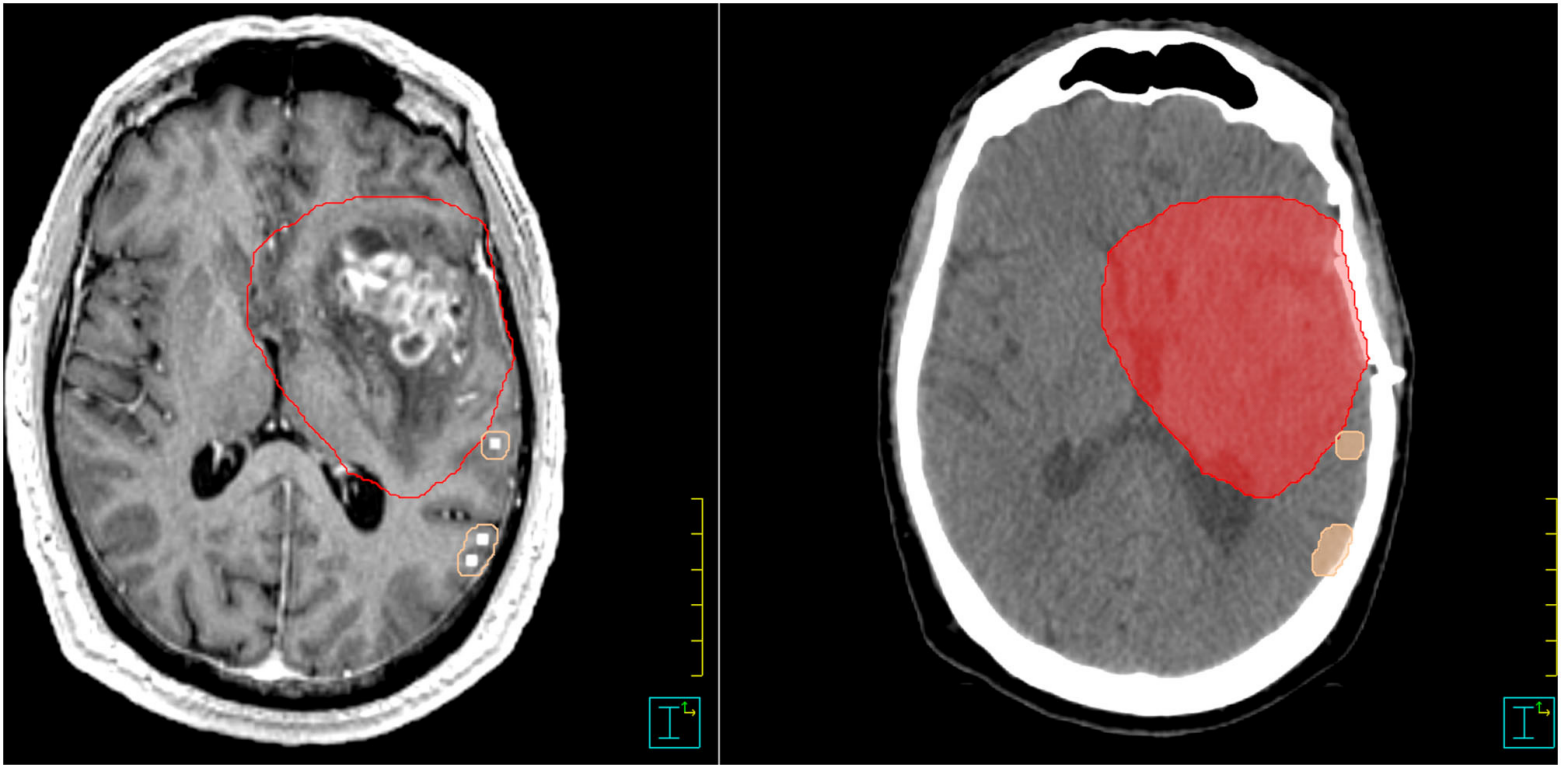
Example for nTMS-mapping and contouring in Pinnacle. nTMS, navigated transcranial magnetic stimulation. PTV = red color; motor cortex = skin color.

For each patient, the nTMS mapping was imported as an additional secondary dataset into the original radiotherapy treatment plan in the Philips Pinnacle TPS V16.2. This image was rigidly co-registered to the primary data set (planning CT) and planning MRI (acquired post-operatively in both T1 MP-RAGE and T2 flair weighting sequence) based on a mutual information algorithm and then manually adjusted until the optimal alignment was achieved. Two radiation physicists and experienced radiation oncologists verified the co-registration independently. The original clinical target volume (CTV), the planning target volume (PTV), and OAR (lens, bulbus oculi, optic nerve, chiasma, medulla, brainstem, and cochlea) for brain irradiation as defined on the planning CT and planning MRI were reviewed, and the additional OARs were contoured: the hippocampi were defined based on T1-weighted planning MRI sequences according to delineation guidelines (42, 43). The nTMS-based motor cortex was contoured by a radiation oncologist by creating a new structure based on delineating the nTMS-positive eloquent points in a brush size slightly larger than the pixel size of the points so that a small margin around the motor-positive points is generated—this is just large enough so that adjacent motor-eloquent points coalesce into a unique structure as shown in Figure 1. No further post-processing of the nTMS-derived OAR was performed.

### Treatment Planning

The original clinical plan for each patient was used, that is an intensity-modulated radiotherapy (IMRT) technique on a Siemens Artiste linear accelerator with 6 MV photon beams. For each patient, four re-optimized treatment plans were created in addition to the original clinically treated plan (“Original”) using the Philips Pinnacle V16.2 TPS (Philips Healthcare, DA Best, Netherlands):

1. a re-optimized plan with sparing of the nTMS-defined motor cortex as an additional planning objective (“Manual Motor”),
2. a re-optimized plan with sparing of the nTMS-defined motor cortex and bilateral hippocampi as additional planning objectives (“Manual M+H”),
3. an automatically optimized plan sparing the nTMS-defined motor cortex (“Auto Motor”), and
4. an automatically optimized plan sparing both the nTMS-defined motor cortex and the bilateral hippocampi, referred to as “Auto M+H.”

The normal plan optimization criteria are given in Table 1. For the manually optimized plans, the first optimization run was carried out using only the PTV and ring constructs; the OAR objectives were only added where the OARs fell close to the PTV and were not adequately spared in the first optimization.

**Table 1:** Standard optimization constraints for the “Original” plans and Auto-Planning plans.

	**Structure**	**Objective**
PTV and dose fall-off	PTV	Uniform dose 60 Gy (100%)
		D_min_ 57 Gy (95%)
		D_max_ 63 Gy
	PTV-Ring 1 (+0 to +5 mm)	D_max_ 57 Gy (95%)
	PTV-Ring 2 (+5 to +8 mm)	D_max_ 54 Gy (90%)
	External without PTV (+8 mm)	D_max_ 48 Gy (80%)
OAR's	Bulbi oculi	D_mean_ <35 Gy
	Lens	D_max_ <5 Gy
	Optic nerve	D_max_ <50 Gy
	Chiasma	D_max_ <50 Gy
	Brainstem	V_54Gy_ <10%
	Medulla	D_max_ <42 Gy
	Cochlea	D_max_ <35 Gy
	Spinal canal	D_max_ <42 Gy
New objectives	nTMS-defined motor cortex	As low as possible
	Hippocampus	As low as possible

*If the PTV overlaps with an OAR or is near to it, an individual objective is required based on a clinical decision by the responsible radiation oncologist*.

*PTV, planning target volume; OAR, organs at risk; nTMS, navigated transcranial magnetic stimulation*.

The Auto-Planning algorithm of Pinnacle is a protocol-based automatic iterative optimization algorithm (39, 44). This algorithm tries to find the most effective balance between target coverage and OAR sparing based on user-defined priorities. Based on the user-defined contours, OAR objectives, and dose prescription for the target, the algorithm generates further optimization “help” structures in consideration of overlaps between PTV and OARs, ring structures for a controlled dose fall-off, and other structures to control target uniformity and dose spillage. Sequential optimization runs are used to best satisfy the user-defined dose objectives. Differently from library-based automatization algorithms, no knowledge of a set of other plans is required and the planning approach closely resembles an experienced manual planner. The advantage of the Auto-Planning algorithm is the iterative nature and the inclusion of additional and automated help structures, which provides more complexity to the optimization than manually achievable. Furthermore, the algorithm does not stop once the pre-set objectives are satisfied, but rather tries to reduce the dose in the OARs below the demanded maximal dose (39, 41, 44).

The “Manual Motor” plan used the same optimization criteria as the “Original” plan and an additional criterion for the motor areas delineated by nTMS exteriorly to the PTV. Since none of the patients had the motor cortex located completely external to the PTV, the goal of this re-optimization was the reduction of the nTMS-based motor cortex mean dose without reducing the coverage of the PTV. The motor cortex dose was pushed as far as possible, accepting up to 0.5 Gy decrease in mean PTV dose.

The aims of the manual motor cortex and hippocampus sparing “Manual M+H” plan were the dose reduction of the motor cortex mean dose and the dose reduction in the hippocampi without reducing the coverage of the PTV. Here too, the optimization was carried out using the same optimization constraints as the “Original” plan adding the new constraints for the motor cortex and hippocampi.

Both the Auto-Planning “AP Motor” plan and the “AP M+H” plan were optimized using the Pinnacle Auto-Planning algorithm. The aims of “AP Motor” and “AP M+H” were the same as the aims of “Manual Motor” and “Manual M+H,” respectively. Note that no automatization was included at the stage of motor cortex delineation, but only in the dose planning step.

The numbers of fields and the allowed segments in all re-optimized plans were equivalent to the used fields and segments of the “Original” plan. The direction of the fields was not changed. The final dose distributions were calculated using a collapsed cone (CC) convolution algorithm on a 2-mm dose grid. All plans were revised by experienced radiation oncologists and radiation physicists and were considered clinically acceptable.

### Plan Evaluation

Different quality parameters were analyzed to evaluate the plan quality.

The Paddick conformity index (CI) (45–47).


(1) $$\begin{array}{r} CI=OR•UR=\frac{TV_{PIV}^{2}}{PIV•TV} \end{array}$$

is the product of the Paddick overdose ratio (OR) and underdose ratio (UR). In the ideal plan, the CI is unity and decreases with decreasing plan quality.

The OR


(2) $$\begin{array}{r} OR=\frac{TV_{PIV}}{PIV} \end{array}$$

is ideally 1, when the PTV volume inside the 95% isodose (TV_PIV_) is the same size as the total volume irradiated with 95% of the total prescription (PIV).

The UR


(3) $$\begin{array}{r} UR=\frac{TV_{PIV}}{TV} \end{array}$$

is ideally 1, when the PTV volume inside the 95% isodose (TV_PIV_) is the same size as the total PTV volume (TV).

The homogeneity index (HI)


(4) $$\begin{array}{r} HI=\frac{PTV_{01\%}-PTV_{99\%}}{PTV_{mean}} \end{array}$$

estimates the dose homogeneity inside the PTV by the ratio of the absolute dose difference (PTV_01%_ dose maximum and PTV_99%_ dose minimum) and the mean dose. In the ideal plan the HI equals 0.

The gradient index (GI)


(5) $$\begin{array}{r} GI=\frac{PIV}{V_{50\%}} \end{array}$$

estimates the steepness of the dose fall-off by the ratio of the prescription isodose volume and the volume of half of this dose (V_50%_).

Besides these parameters, the maximum and the mean dose of the PTV and the OARs, especially the motor cortex and the hippocampi, are determined. For the motor cortex and the ipsilateral hippocampus, the intersection between the respective OAR and the different isodoses is analyzed.

### Statistical Analyses

By an in-house Pinnacle script, the dose-volume histogram (DVH) values of each OAR, PTV, and motor cortex were exported into a comma separated variables (CSV) table. All calculations and statistical analyses were performed with MATLAB R2019b. A normal distribution could not be presumed, so Wilcoxon's signed-rank test of paired data with Bonferroni correction was used, reducing the level of significance to 0.005.

## Results

**Figure 2:**
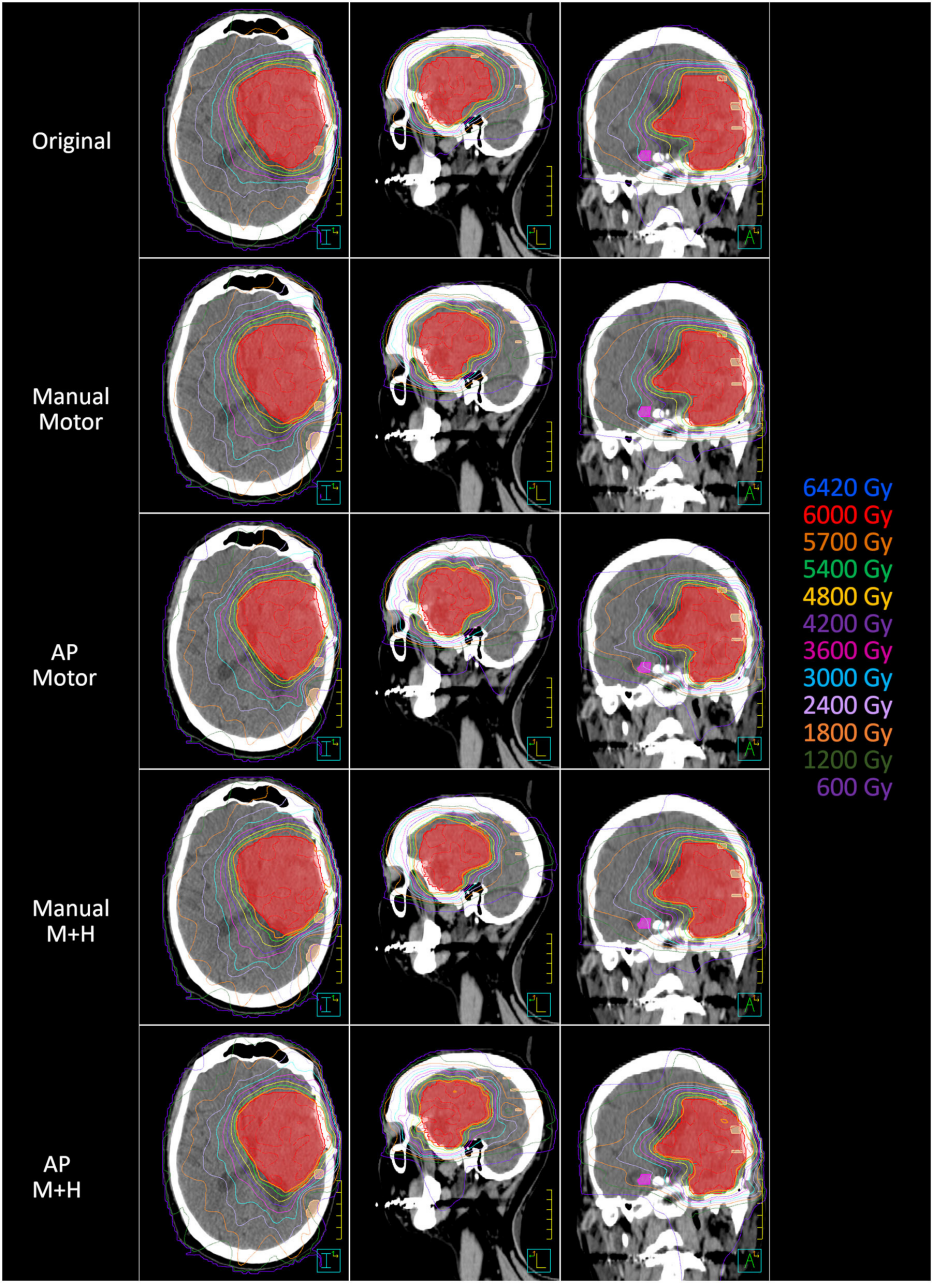
Example of the dose distribution in the planning CT (axial, sagittal, and coronal axis) (left, middle, and right). PTV = red color, motor cortex = skin color, contralateral hippocampus = pink color.

**Figure 3:**
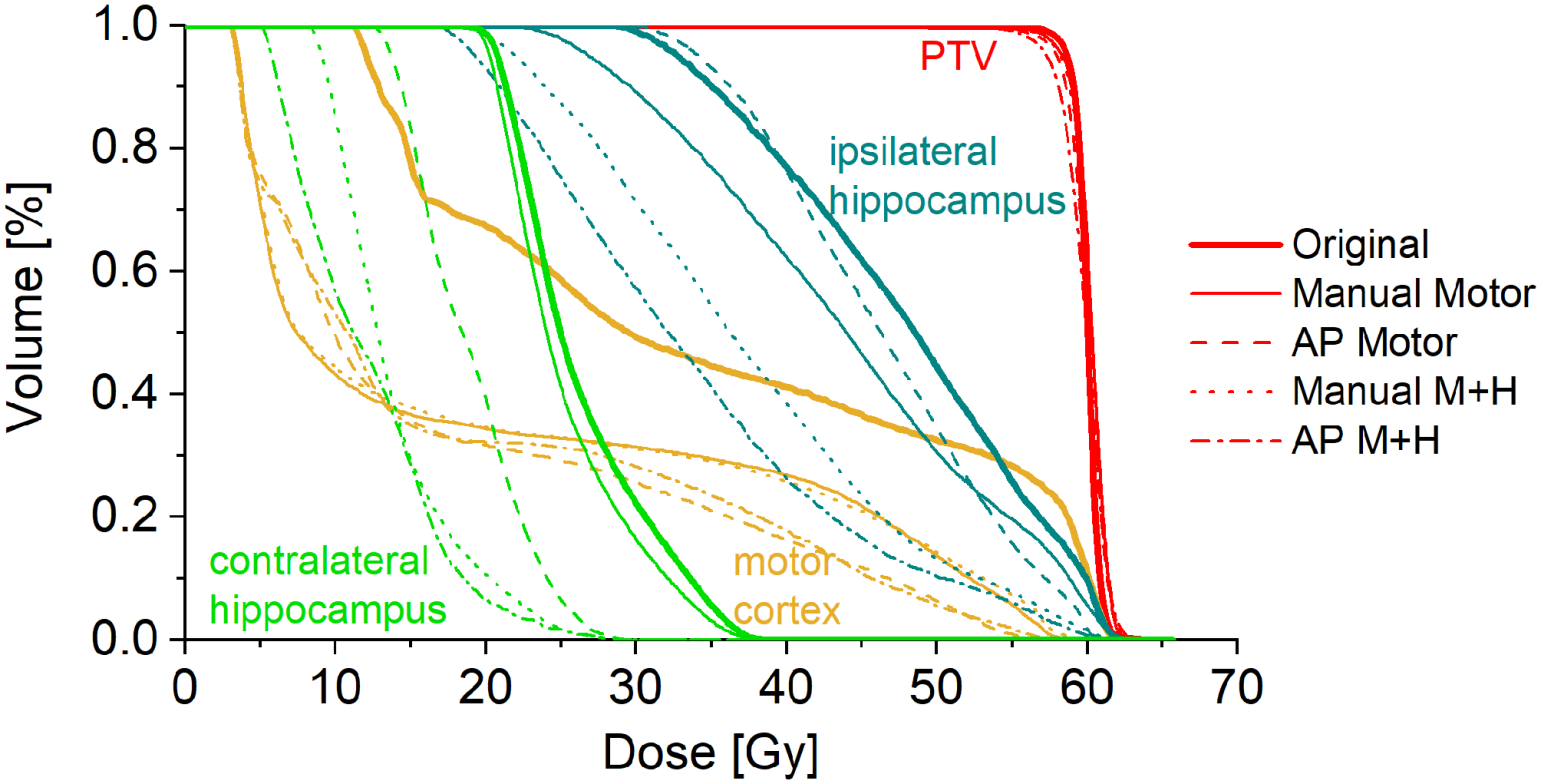
Dose-volume histogram for the example patient in Figure 1.

A total of 24 patients received radiation therapy and surgical resection at the authors' department. Only patients with a prescription of 30 fractions of 2 Gy were included to allow for a more homogeneous collective and more valid dosimetric comparison. Also, patients whose motor cortex was located to more than 95% inside the PTV were excluded from this study because a dose reduction was not reasonable. For the remaining 10 patients, the four re-optimized plans “Manual Motor,” “AP Motor,” “Manual M+H,” and “AP M+H” were clinically acceptable. In Table 2, the distribution of the overlap of the motor cortex with the PTV is presented, while in Figure 2, an example for the resulting isodose distributions with the related DVH (Figure 3) is shown. In Supplementary Table 1, the metrics for plan quality and the dose to the PTV motor cortex and OARs are given.

**Table 2:** Overview of the overlap of motor cortex and PTV.

**Percent of motor cortex in PTV**	**Number of patients**
<24	3
25–49	1
50–74	1
75–94	5

*PTV, planning target volume*.

### PTV Coverage and Plan Quality Metrics

**Figure 4:**
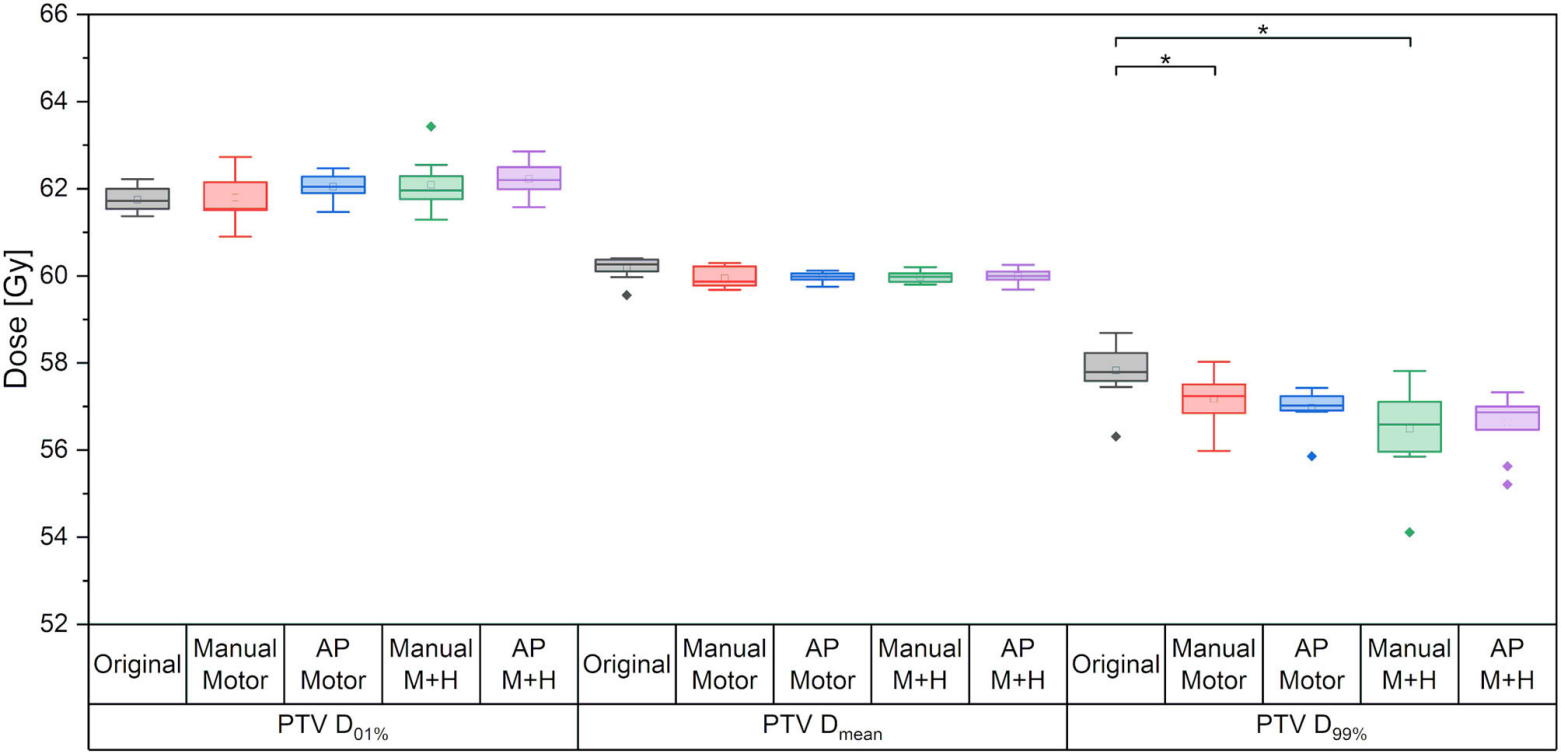
Maximum dose (D_01%_), mean dose and minimum dose (D99%) to the PTV. Line = median value, square = mean value, diamond = outliers, star = statistically significant at *p* < 0.005.

For the maximum dose and the mean dose to the PTV, there was no statistically significant difference between the five kinds of plans (Figure 4). The minimum dose decreased slightly for all re-optimized plans, but only reached statistical significance for the manual plans. From a clinical perspective, this decrease was deemed adequate and would not have impaired plan acceptability.

The brain without PTV received a slightly decreased mean dose (<1 Gy) in the re-optimized plans, which was statistically significant for the manually optimized plans, while the maximum dose remained almost the same.

**Figure 5:**
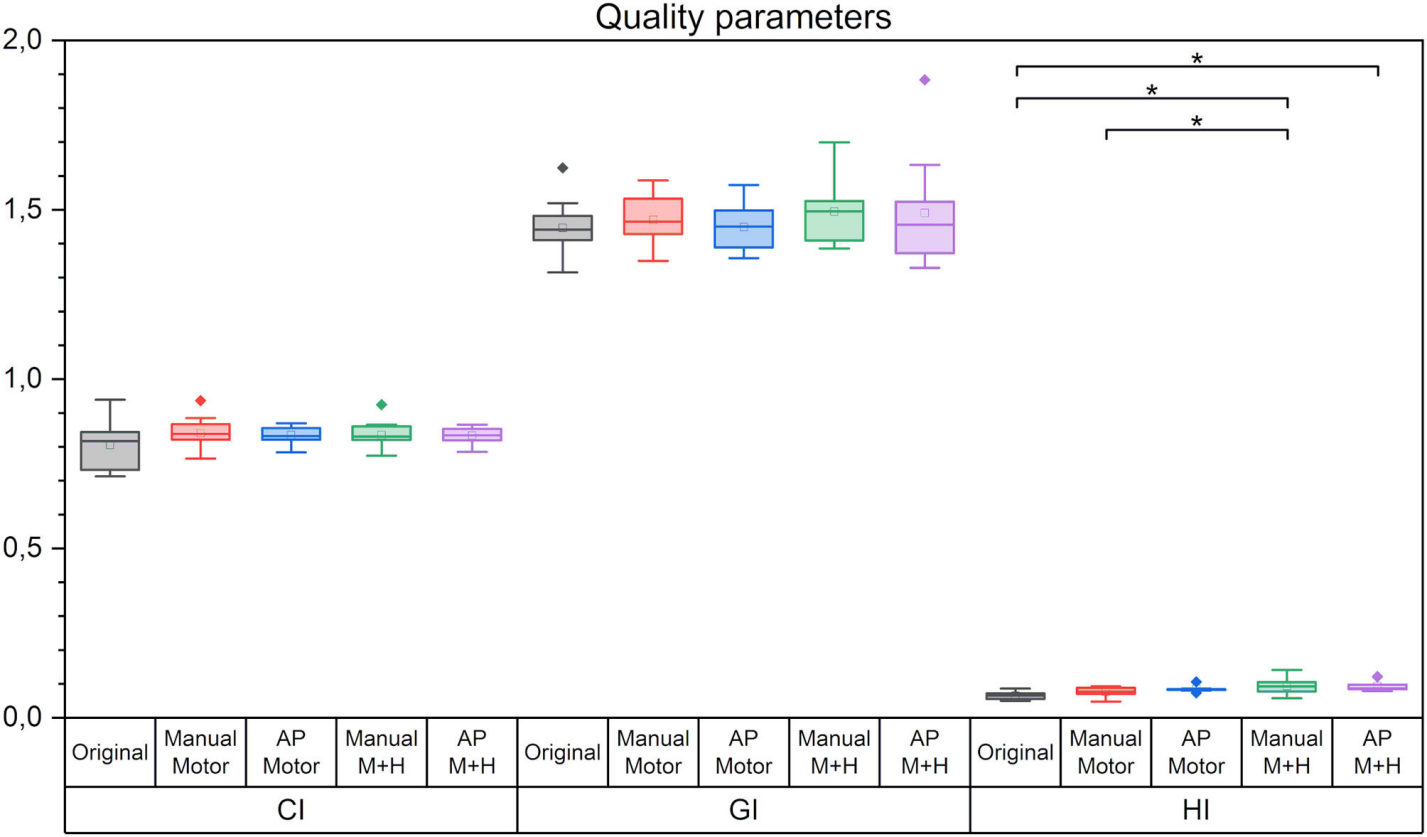
Quality parameters conformity index (CI), gradient index (GI), and homogeneity index (HI). Line = median value, square = mean value, diamond = outliers. There were no statistically significant differences between the planning scenarios.

For all plan quality parameters, there were hardly any significant differences between the five plans (Figure 5). The CI, OR, HI, and the GI appear slightly higher while the UR decreased; however, only the increase in HI for the motor cortex and hippocampus sparing plans is statistically significant and would not have resulted in the clinical rejection of the plans.

### Organs at Risk

The OAR originally included in the clinical plans showed no significant or clinically relevant increase of the maximum or mean dose. The dose of all OARs in the “Original” plans was already below the clinical critical value. OAR near the hippocampi, such as brainstem or medulla, had a significant decrease in the maximum dose (up to 11 Gy for the brainstem in the “AP M+H” plan) and mean dose (up to 5.7 Gy for the brainstem in the “AP M+H” plan) for all re-optimized plans except for the “Manual Motor” plans, which did not optimize hippocampus dose. For most of the OARs, a significant further reduction of the maximum and mean doses was achieved by the Auto-Planning plans. Only if the doses in the “Original” plans were already very low (i.e., lenses located very far from the PTV), no significant reduction could be found.

### Motor Cortex Sparing

**Figure 6:**
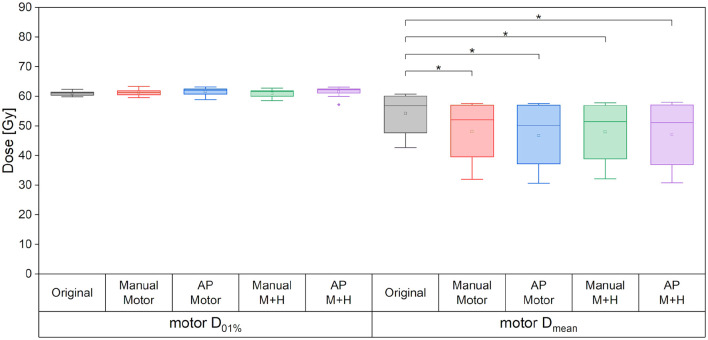
Maximum and mean dose to the motor cortex. Line = median value, square = mean value, diamond = outliers, star = statistically significant at *p* < 0.005.

**Figure 7:**
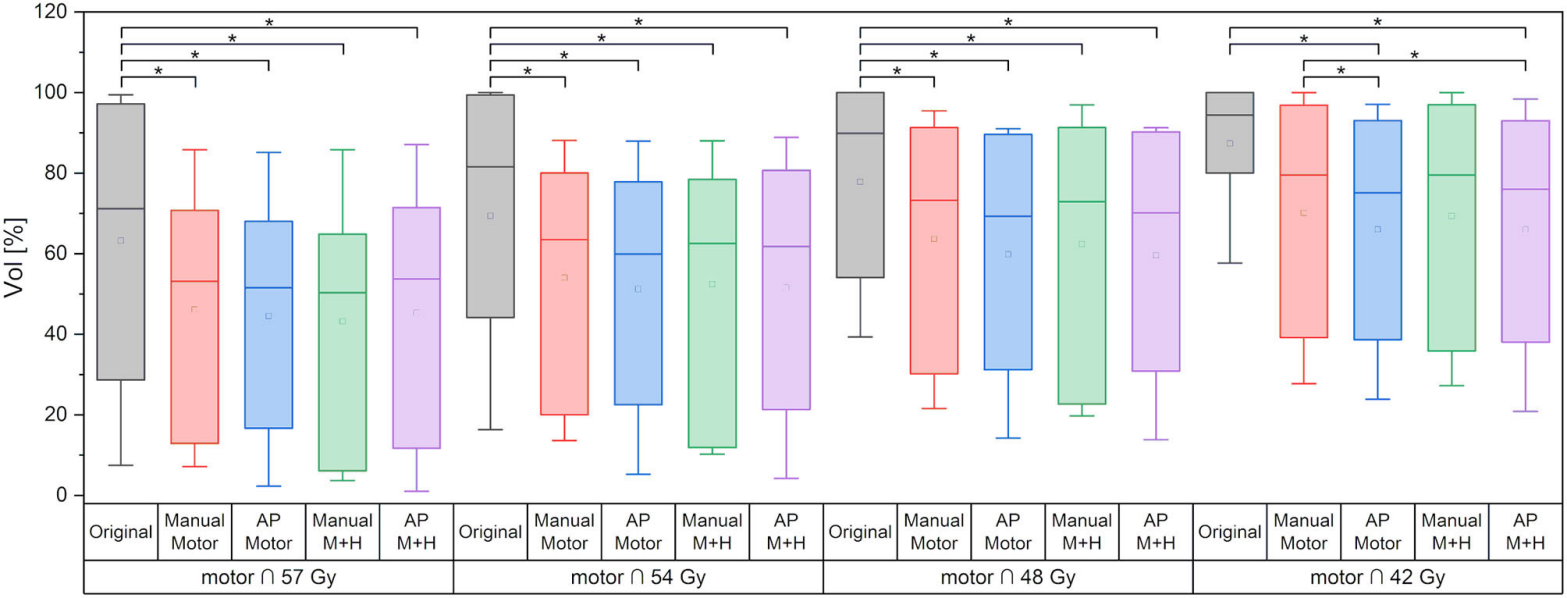
Overlap of motor cortex and isodose levels. Line = median value, square = mean value, diamond = outliers, star = statistically significant at *p* < 0.005.

For the motor cortex, a significant reduction in the mean dose for all four re-optimized plans could be achieved [from 54.3 ± 6.7 Gy (“Original”) to 48.2 ± 10.2 Gy (“Manual Motor”), 46.8 ± 11.2 Gy (“AP Motor”), 48.1 ± 10.2 Gy (“Manual Motor+Hipp”), and 47.1 ± 11.4 Gy (“AP Motor+Hipp”), Figure 6], while there was no difference in the maximum dose (near the prescribed dose since this contour overlapped with the PTV). The overlap between the motor cortex and the 57, 54, 48, and 42 Gy isodoses is significantly reduced for all re-optimized plans (Figure 7). The improvement for the Auto-Planning plans was a little higher than for the manually optimized plans, but without reaching statistical significance. For the other isodoses, no significant changes occurred, given that the overlap for the lower doses with the motor cortex approached 100%.

**Figure 8:**
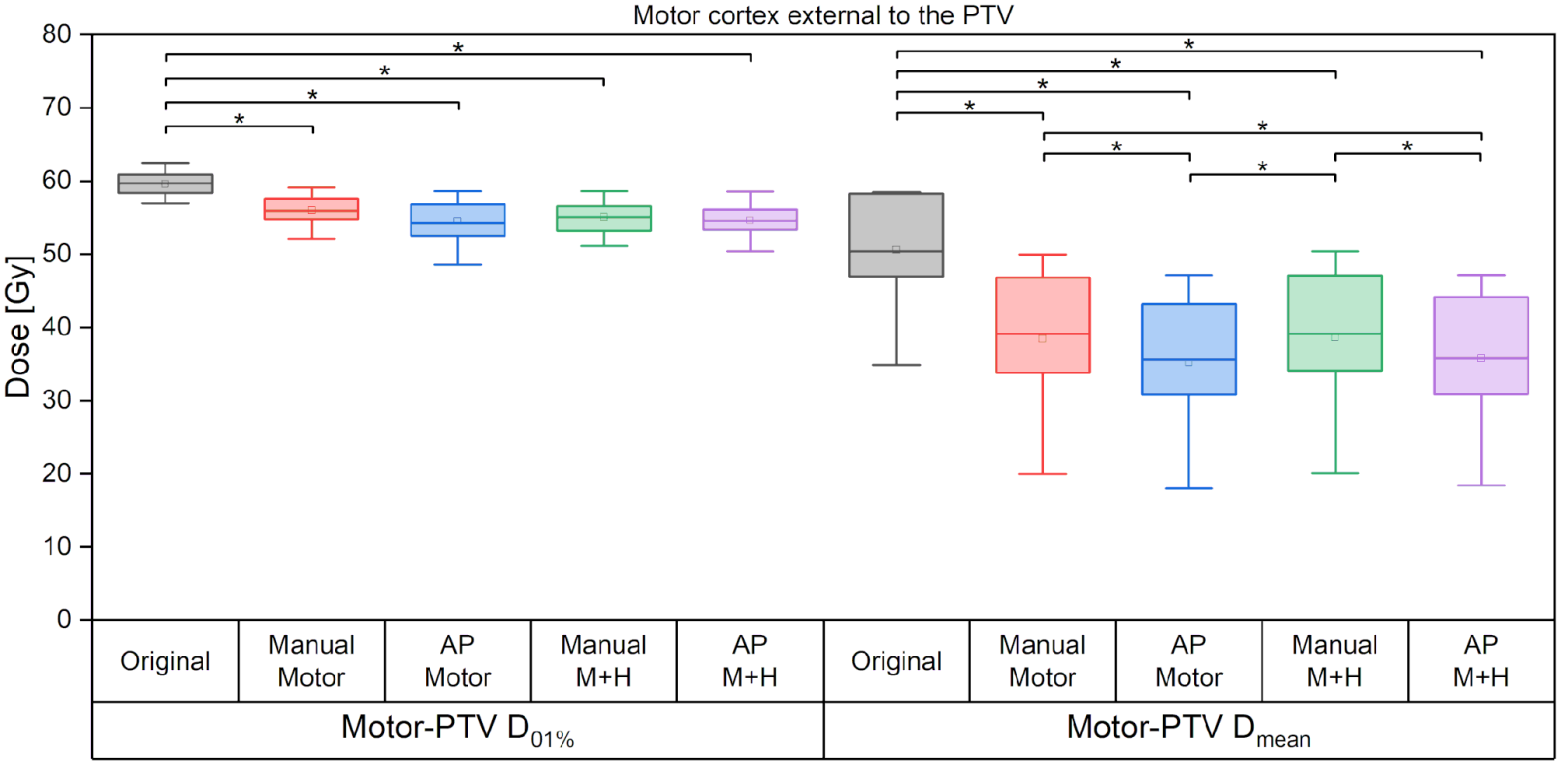
Maximum and mean dose to the motor cortex. Line = median value, square = mean value, diamond = outliers, star = statistically significant at *p* < 0.005.

If only the motor cortex outside the PTV is analyzed, then there is a significant reduction of the maximum dose and mean dose for all re-optimized plans (Figure 8). The maximum dose is reduced by around 3 Gy for the manually optimized plans and around 4 Gy for the Auto-Planning plans. The mean dose is reduced by 12 Gy for the manual plans and by 13 Gy for the Auto-Planning plans, and the difference between the manual and Auto-Planning plans is significant for both optimization goals.

### Hippocampus Sparing

**Figure 9:**
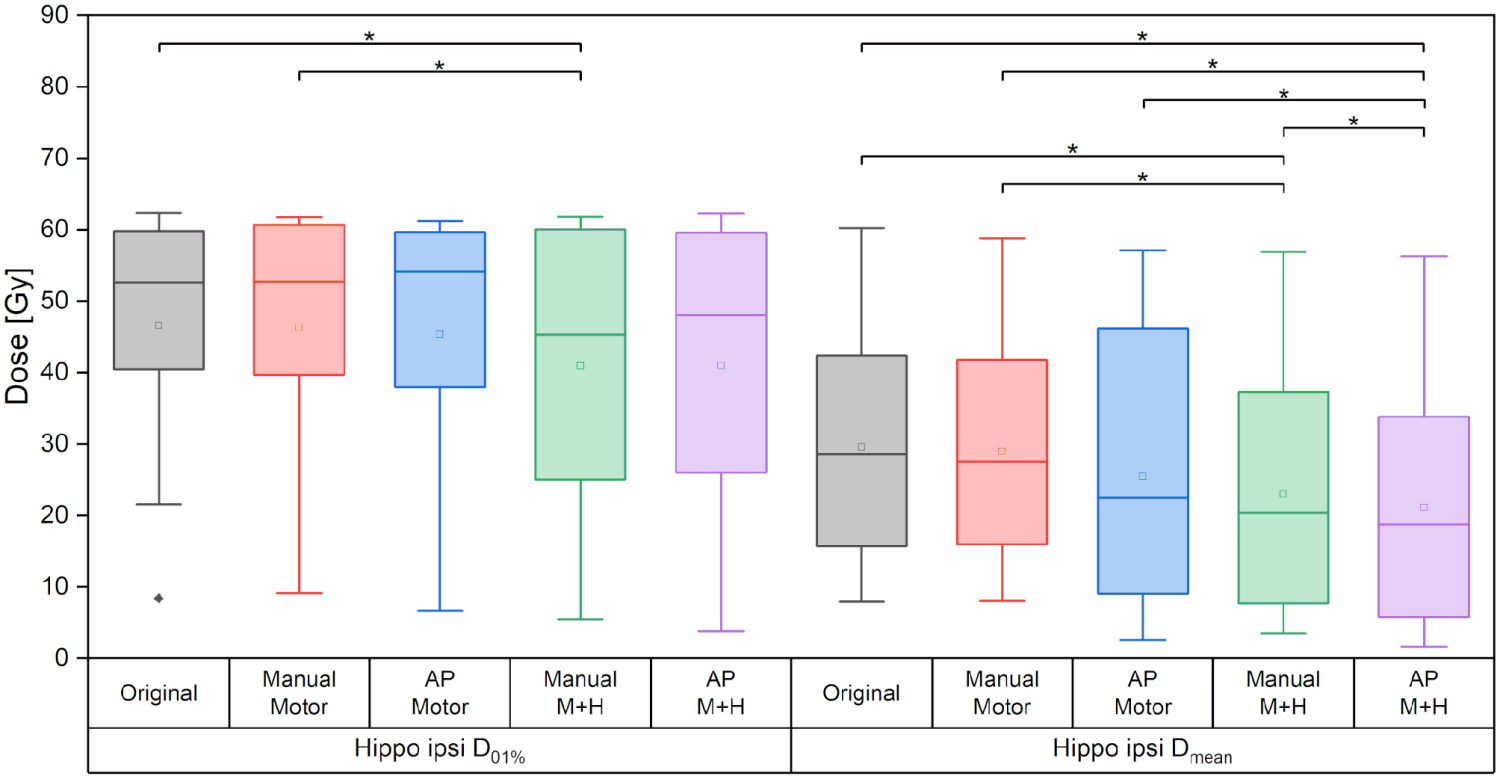
Maximum and mean dose of the ipsilateral hippocampus. Line = median value, square = mean value, diamond = outliers, star = statistically significant *p* < 0.005.

Taking the average of the maximum ipsilateral hippocampus dose over the collective of patients, a reduction of more than 5.5 Gy for the hippocampus-sparing plans could be seen (“Original” 46.6 ± 18.1 Gy, “Manual Motor” 46.3 ± 18.0 Gy, “AP Motor” 45.4 ± 19.7 Gy, vs. “Manual M+H” 40.9 ± 19.6 Gy, “AP M+H” 40.9 ± 20.9 Gy, Figure 9); however, given the large variability in maximum hippocampus doses over the collective statistical significance could only be proven between the “Manual M+H” plan and the “Original” and “Manual Motor” plans. For the mean doses to the ipsilateral hippocampus, a significant improvement was observed in all re-optimized plans. The reduction of the only motor sparing plans was not as large as the reduction of the motor and hippocampi sparing plans, which was statistically significant (“Original” 29.6 ± 18.3 Gy, “Manual Motor” 29.0 ± 17.3 Gy, “AP Motor” 25.4 ± 20.2 Gy, “Manual M+H” 23.0 ± 18.2 Gy, and “AP M+H” 21.1 ± 18.2 Gy). Meanwhile the Auto-Planning plans attained an even higher reduction than the manually optimized ones so that the “AP M+H” plan offered the largest improvement (statistically significant in comparison with all other plans).

**Figure 10:**
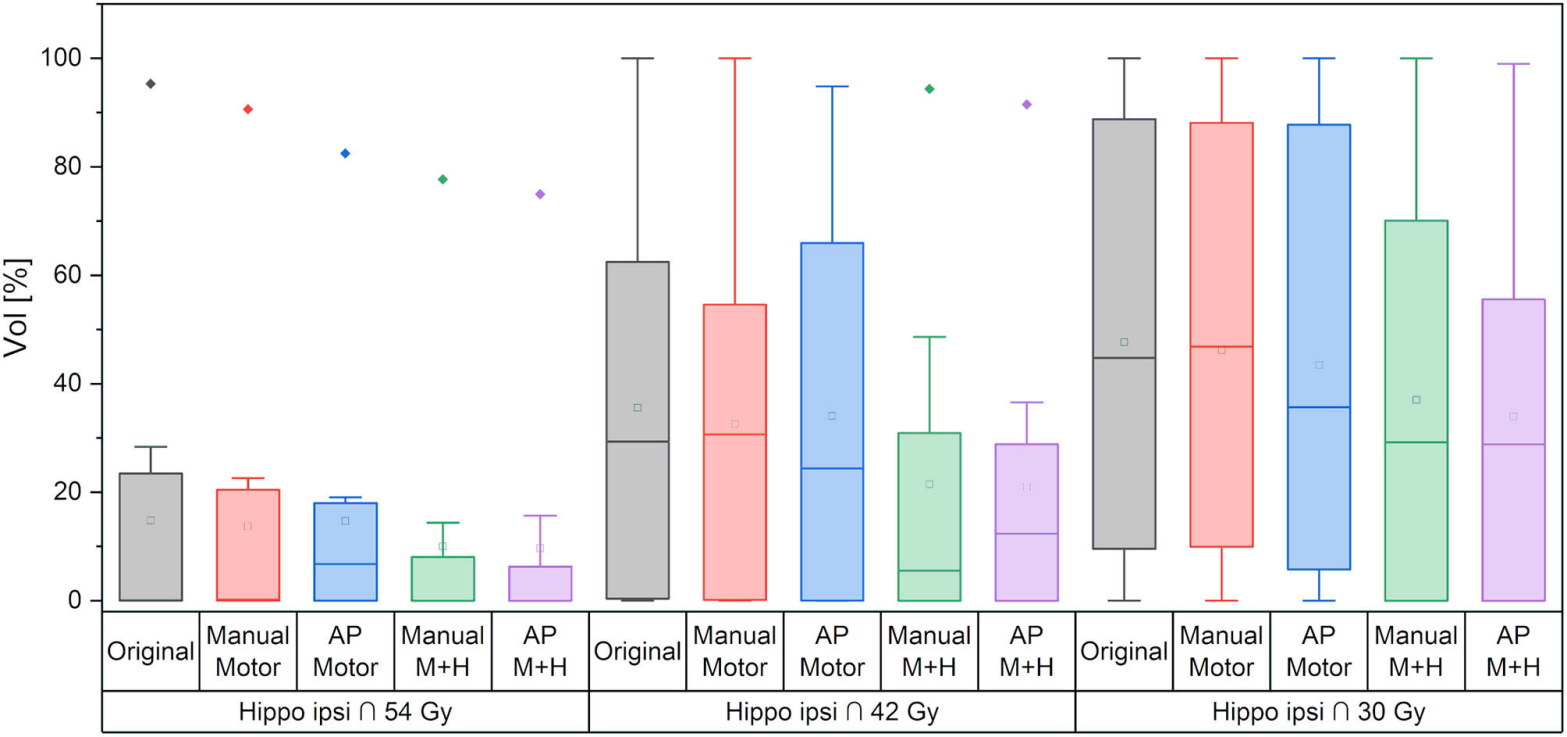
Overlap of ipsilateral hippocampus and isodose levels. Line = median value, square = mean value, diamond = outliers, star = statistically significant *p* < 0.005.

Regarding the spatial overlap between the ipsilateral hippocampus and isodose levels, there were no significant differences between the plans, although it can be seen from Figure 10 that the intersection between the hippocampus and higher isodose levels has decreased for the plans with motor cortex and hippocampus sparing.

**Figure 11:**
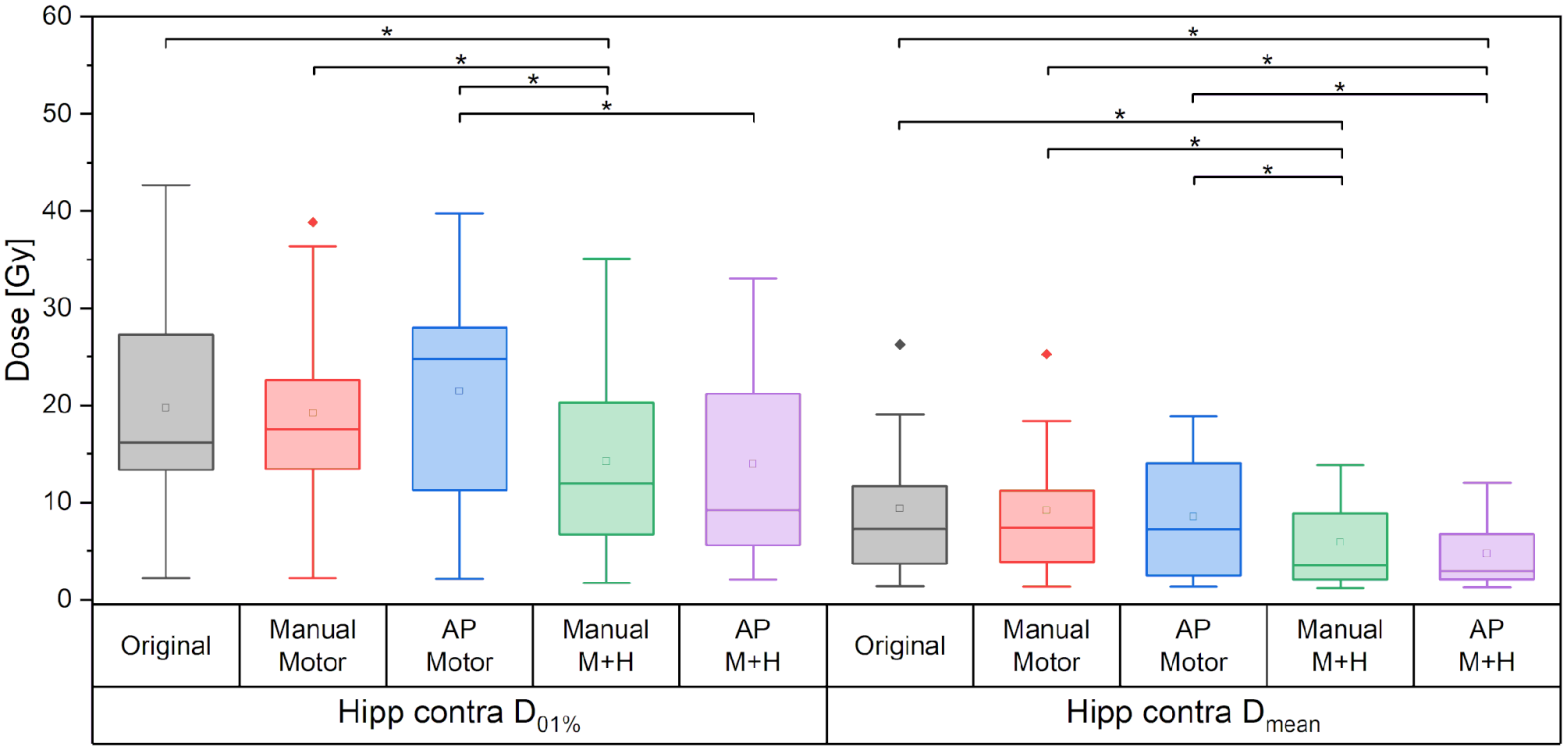
Maximum and mean dose to the contralateral hippocampus. Line = median value, square = mean value, diamond = outliers, star = statistically significant *p* < 0.005.

For the motor cortex and hippocampi sparing plans, the maximum dose of the contralateral hippocampus was significantly reduced by more than 5.5 Gy and the mean dose by about 3.5 Gy as compared with the “Original” plan (Figure 11).

### Plan Efficiency and Modulation

**Figure 12:**
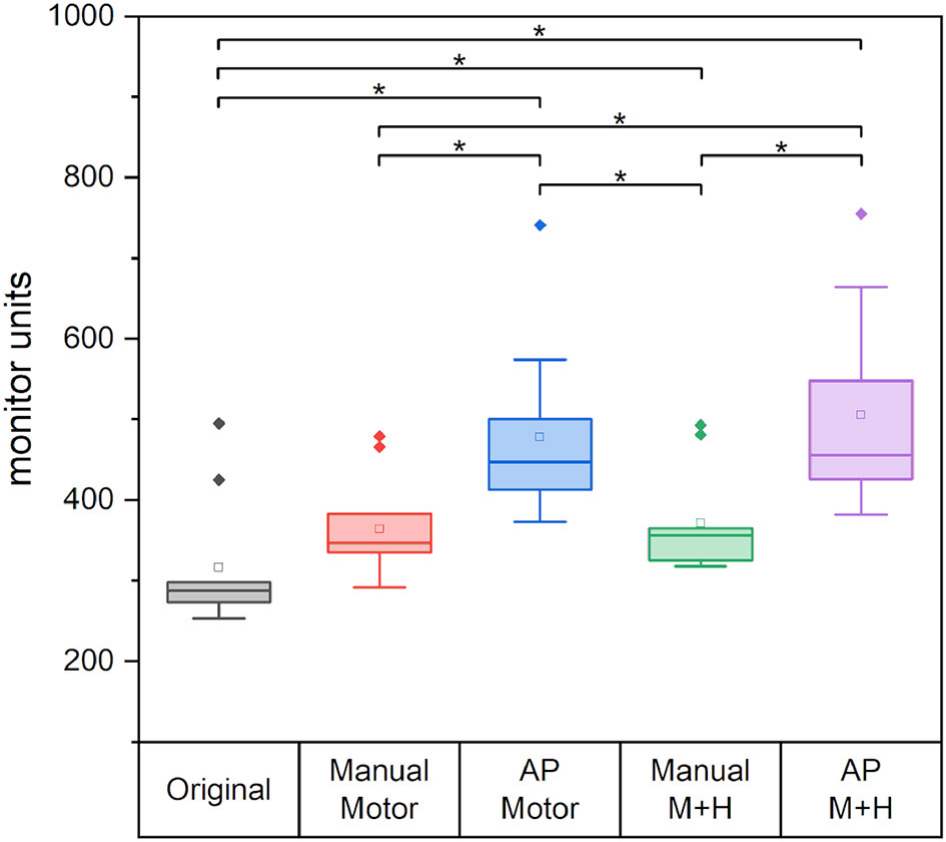
The required monitor units for the different plans. Line = median value, square = mean value, diamond = outliers, star = statistically significant *p* < 0.005.

Figure 12 gives the required monitor units (MU) of the planning scenarios. For all re-optimized plans, the monitor units are significantly increased except for the “Manual Motor” plan for which no significance could be proven. The increase for the plans which spare motor cortex and hippocampi, is significantly larger than for the plans which only consider the motor cortex. Also, the plans optimized with Auto-Planning require significantly higher monitor units, i.e., higher modulation of the beams.

## Discussion

Although we had only a small number of patients available for analysis, this was a homogeneous collective with very comparable clinical scenarios. For this cohort, a total of 50 treatment plans were compared, which were created in a reproducible manner and revised by an attending radiation oncologist and a senior medical physicist. This study could show that combined sparing of the nTMS-defined motor cortex and the hippocampus is feasible in the radiotherapeutic treatment of patients with GBM without compromising target coverage, plan quality metrics, or sparing of traditional OAR, even in a collective of patients where the motor cortex was partially included in the PTV.

The main goal, which was the sparing of the motor cortex and the hippocampi, could be achieved. The motor cortex mean dose was reduced by more than 10% (>6 Gy) in the whole nTMS-defined cortex and by up to 25% (>12 Gy) for the part of the motor cortex located outside of the PTV. A reduction of the maximum dose could expectedly not be achieved because the cortex overlaps largely with the PTV. However, the maximum dose of the motor cortex outside the PTV was significantly reduced for all plans by more than 3.5 Gy. Furthermore, the overlap of the motor cortex with high-dose isodoses could be significantly decreased.

For the ipsilateral and contralateral hippocampus, a reduction in the mean dose of over 20% (>6 Gy) and 40% (>4.6 Gy), respectively, could be achieved. The maximum doses were decreased significantly by over 10% (>5 Gy) and 20% (>5 Gy) for the ipsilateral and contralateral hippocampus, respectively.

### Benefits of Automated Planning

Using the Auto-Planning algorithm, the motor cortex located outside the PTV could be better protected than in the manual plans; all other objectives (complete motor cortex, ipsilateral, and contralateral hippocampi) were equally satisfied by the manual and Auto-planning scenarios with no significant differences.

While several studies for different tumor entities have shown that Auto-Planning resulted in a better plan quality than manual plans [e.g., (38, 48)], this is not supported in our study collective. The PTV coverage and the plan quality defined through the plan quality parameters were the same for the “Original” plan and both kinds of planning. The similar plan quality indicates that the plan quality of the manual plans was good. However, the spread of the mean and maximum dose and the quality factors were smaller for the Auto-Planning. In addition, the variation in dose to the healthy brain without PTV is smaller for Auto-Planning than manual. While only a reduction in measures of statistical spread (such as the interquartile range as seen in the box plots above) precludes a statistically significant difference between the manual and automated plans, this may point to better reproducibility and stability of the Auto-Planning results.

In contrast, the variation for the motor cortex dose is higher for Auto-Planning than for the manual plans. This effect could be due to the overlap of the motor cortex and the PTV. The conflicting objectives were not always achievable, which was a problem for the Auto-Planning algorithm, that could more consistently be solved manually by the informed decision of the planner. Nevertheless, on the average, the Auto-Planning performed slightly better.

Due to the different optimization parameters, the Auto-Planning reduced the dose for almost all other OARs significantly even when they were already below the constraints. This is one expressed aim of the Auto-Planning algorithm, as it strives to satisfy all the objectives and if possible to undercut them (41). The manual plans only aimed to reduce the dose in the other OARs if they were too high from a clinical point of view and ceased the efforts when the clinical objectives were reached. The second advantage of Auto-Planning is that one optimization is enough to produce a clinically acceptable plan, while for the manual plans, many optimization runs were normally needed. Hence, the clinical workflow was greatly relieved by knowing that an adequate plan could be reliably be created with the known set of objectives. In the clinical reality, this would also allow for a less experienced planner to create high-quality plans based on the Auto-Planning template, whereas an experienced planner invested considerable time and effort into the manual optimizations.

However, the good performance and partly even better sparing of the Auto-Planning scenarios come at the cost of higher intensity modulation expressed in the plan MU. While the manual plan with hippocampi sparing increased significantly by around 60 MU (−2 MU to +277 MU) when compared with the original plan, which amounts to about 19%, the increase for the Auto-Planning plans was significantly higher with over 185 MU (almost 60% higher than in the original plans, “AP Motor”: +75 MU to +277 MU and “AP M+H”: +84 MU to +367 MU). Such a large increase in irradiated dose will contribute to higher head scatter and leakage radiation, which is not precisely accounted for in the treatment plans and may be relevant from the perspective of radiation protection.

### Comparison With Previous Studies on nTMS-Based Motor Cortex Optimization

A small number of studies have systematically evaluated the motor cortex as an optimization objective in radiotherapy treatment planning. As early as 2013, Conti et al. (23) used nTMS and functional MRI data and tractography of the motor and language system to optimize dose distributions in CyberKnife radiosurgery of 25 patients, achieving a 17% reduction in dose to functional areas. Focusing on the nTMS-defined motor cortex, Tokarev et al. (25) could reduce the maximum dose by up to 17% (average 6%) and the >12 Gy-volume by 2–78% (average 35.2%) in eight patients planned using GammaKnife. For linear-accelerator-based treatment of brain metastases, Schwendner et al. (22) reported a mean dose reduction to the nTMS-based motor cortex of 18% for a collective of 30 patients planned with VMAT, which corresponded to our own results for patients with metastases treated with IMRT, non-coplanar arcs, or static beams [sparing of about 30% in mean dose, but depending on planning technique and distance between the lesion and the motor cortex, (26)].

Only one study hitherto considered patients with malignant gliomas, which present a rather different patient collective with different needs for planning and optimization. In general, much larger target volumes are treated with modulated techniques in standard fractionations to 60 Gy or more, resulting in different necessities and possibilities for plan optimization. Diehl et al. (21) re-optimized plans for 30 patients with high-grade gliomas setting an objective of 45 Gy maximum dose to the nTMS-defined motor cortex outside the PTV [prescription 59.4 Gy/70 Gy in PTV/simultaneous integrated boost (SiB)]. The resulting mean dose to the motor maps was reduced by 12.8%, and the volumes of the motor maps receiving > 45 Gy decreased by 11.3%, without a relevant decrement in overall plan quality. We chose a somewhat modified approach in setting the motor map objectives as low as achievable for each patient before compromising PTV coverage. Still, a similar decrease in mean motor cortex dose (10%, 6 Gy) and a significant decrease in the overlap with the high dose isodoses are obtained. In particular, a further slight but statistically significant improvement in the overlap volumes could still be achieved using the Auto-Planning modality. Furthermore, for all patients, we could obtain the motor cortex dose protection not only without compromising PTV coverage and the sparing of other OAR but also when including additional constraints on the bilateral hippocampi.

Beyond the dosimetric optimization, such as the nTMS information into radiotherapy treatment planning, may offer further benefits. Picht et al. (20), presenting a mixed collective of 11 patients with metastases, meningiomas, and AMVs, evaluated whether the nTMS information on the motor cortex influenced patient counseling, contouring, and other treatment decisions from indication to dose prescription and optimization. All these factors except for the contouring were found to be at least somewhat influenced by the additional information in a large percentage of the patients. It can thus be presumed that the inclusion of nTMS information at an earlier stage than merely in dose optimization can provide further benefits for informed decisions.

### Clinical Benefits of the Motor Cortex and Hippocampus Protection

In how far these dosimetric improvements can be translated into clinical endpoints is yet uncertain. First of all, the clinical effects of radiotherapy may only become evident after some latency and may hence not occur within the remaining lifetime of patients with highly malignant glioma. In this case, an improvement in quality of life would not be achieved. However, several effects of ionizing radiation on the brain have been observed to occur within a time span of weeks to months, such as attention deficit and short-term memory loss (49, 50), or by >6 months after radiotherapy [general cognitive impairment and dementia in brain tumor survivors (50)]. In these patients, preservation of quality of life will be of paramount importance, if this can be achieved.

While motor skills have been reported to be decreased after radiotherapy (18, 19, 51), only little information is available on possible dose thresholds. In addition to the influence on motor skills, Peiffer et al. (12) found that the volume of the precentral gyrus covered by >40 Gy is a significant predictor for verbal immediate recall and trail making test performance, so an improvement in this value might translate into measurable cognitive improvement of the patients. In our paper, a significant reduction in the volume of the motor cortex overlapping with the high isodose levels (above 48 Gy and higher) could be achieved, and some sparing of lower isodose coverage was also feasible.

Regarding the hippocampus, Kim et al. (7) found impaired verbal learning performance for patients receiving a mean left hippocampus dose of between 38 and 43 Gy as compared with those receiving 11–12 Gy. While there was considerable variation in mean hippocampus dose in our patient collective, the hippocampus-optimized plans could reduce the dose to the contralateral hippocampus to <14 Gy in the manual and no more than 12 Gy in the Auto-Planning plans for all patients in our collective. The ipsilateral hippocampus mean dose ranged between 8 and 60 Gy (mean 30 Gy) in the original plans and could be reduced by 6.5 and 8.5 Gy in the manual and Auto-Planning optimizations so that it can be expected that some patients would be represented directly on the slope of a dose-response curve and hence benefit from dose reduction. Similarly, the maximum hippocampus dose varied greatly with distance from the PTV; however, it could still be reduced by about 5 Gy, which may be relevant for those patients close to a threshold dose of ca. 12 Gy, which was observed by Tsai et al. (8) to correlate with impaired verbal memory. Summing up the tenuous evidence on dose effects available so far, some tangible clinical and cognitive improvements may be achieved at least for a subset of the patients receiving radiotherapy. Most importantly, this dose sparing comes at no cost in plan quality regarding either PTV coverage and hence prognosis or sparing of “traditional” OAR. Therefore, to our opinion even a hypothetical and plausible – though as yet unproven – improvement in motor and the cognitive outcome would warrant the additional care in dose optimization.

## Conclusion

Simultaneous dose reduction in the motor cortex and hippocampi is feasible while the PTV coverage, plan quality parameters, and sparing of other OAR are maintained or improved. The overall mean dose reduction to the nTMS-defined motor cortex was up to 10% (6 Gy), while the mean dose to the ipsilateral hippocampus was reduced by more than 20% (6 Gy) and the contralateral by more than 40% (4 Gy). Manual optimization and Auto-Planning achieved considerable dose protection, with slightly better performance of the Auto-Planning algorithms for most of the OAR coming at the cost of considerably higher dose modulation. No apparent disadvantages due to sparing the nTMS-defined motor cortex and hippocampi were found. Consequently, standardized sparing of these structures may be recommended for the clinical routine while the effects for the quality of life, motor function, and cognitive performance need to be determined in future studies.

## Data Availability Statement

The raw data supporting the conclusions of this article will be made available by the authors, without undue reservation.

## Ethics Statement

Ethical review and approval was not required for the study on human participants in accordance with the local legislation and institutional requirements. The patients/participants provided their written informed consent to participate in this study.

## Author Contributions

YD designed the study concept. JO recruited the patients and was responsible for the surgical treatment of the patients and CR was responsible for the radiooncological treatment. Patient datasets were retrieved from the data base and retrospectively revised by YD. Import into the planning system and rigid registration with the planning CT was performed by YD and MS. PM and YD contoured the organs at risk. MS created the re-optimized radiotherapy treatment plans and performed the plan evaluation and statistical analysis. PM and FN evaluated the treatment plans. MS and YD wrote the manuscript and prepared the figures and tables. All authors were involved in the interpretation of the results and contributed to the article and approved the submitted version.

## Conflict of Interest

The authors declare that the research was conducted in the absence of any commercial or financial relationships that could be construed as a potential conflict of interest.

## Publisher's Note

All claims expressed in this article are solely those of the authors and do not necessarily represent those of their affiliated organizations, or those of the publisher, the editors and the reviewers. Any product that may be evaluated in this article, or claim that may be made by its manufacturer, is not guaranteed or endorsed by the publisher.
