# Supplementary material for: Automatic Radiotherapy Planning for Glioblastoma Radiotherapy With Sparing of the Hippocampus and nTMS-Defined Motor Cortex
Source: Front Neurol. 2022 Jan 14;12:787140. doi: 10.3389/fneur.2021.787140 (PMC8795623; doi:10.3389/fneur.2021.787140)
Supplement: Supplementary file 1 [file Table_1.docx]

Supplementary Table 1:
Plan evaluation using metrics for plan quality, PTV coverage, and dose to organs at risk. Average ± standard deviation (min-max); p-value in bold indicates statistical significance (after Bonferroni correction). Bulbi oculi are not shown, because the dose is in all cases below the clinically relevant threshold and not statistically significant. Abbreviations: CI = conformity index; OR = overdose ratio; UR = underdose ratio; HI = homogeneity index; GI = gradient index

|  | **Original**  **(1)** | **Manual Motor (2)** | **AP Motor (3)** | **Manual M+H (4)** | **AP M+H (5)** | **p (1)-(2)** | **p (1)-(3)** | **p (1)-(4)** | **p (1)-(5)** | **p (2)-(3)** | **p (2)-(4)** | **p (2)-(5)** | **p (3)-(4)** | **P (3)-(5)** | **P (4)-(5)** |
| --- | --- | --- | --- | --- | --- | --- | --- | --- | --- | --- | --- | --- | --- | --- | --- |
| PTV |  |  |  |  |  |  |  |  |  |  |  |  |  |  |  |
| D_01%_ [Gy] | 61.755 ± 0.282 (61.37-62.22) | 61.796 ± 0.563 (60.90-62.73) | 62.046 ± 0.286 (61.47-62.47) | 62.087 ± 0.590 (61.29-63.43) | 62.225 ± 0.362 (61.58-62.86) | 0.750 | 0.027 | 0.131 | 0.014 | 0.287 | 0.064 | 0.014 | 0.904 | 0.266 | 0.432 |
| D_mean_ [Gy] | 60.187 ± 0.257 (59.560-60.403) | 59.945 ± 0.226 (59.681-60.294) | 59.968 ± 0.118 (59.754-60.122) | 59.982 ± 0.122 (59.802-60.199) | 59.999 ± 0.174 (59.686-60.251) | 0.049 | 0.020 | 0.084 | 0.084 | 0.846 | 0.432 | 0.432 | 0.770 | 0.557 | 0.625 |
| D_99%_ [Gy] | 57.831 ± 0.684 (56.31-58.69) | 57.168 ± 0.605 (55.98-58.03) | 56.966 ± 0.424 (55.86-57.43) | 56.490 ± 1.060 (54.11-57.82) | 56.634 ± 0.687 (55.21-57.33) | **0.002** | 0.014 | **0.002** | 0.010 | 0.232 | 0.020 | 0.084 | 0.275 | 0.039 | 1.000 |
| CI | 0.805 ± 0.072 (0.713-0.939) | 0.841 ± 0.051 (0.765-0.937) | 0.835 ± 0.025 (0.783-0.870) | 0.835 ± 0.043 (0.774-0.924) | 0.834 ± 0.023 (0.785-0.866) | 0.020 | 0.232 | 0.049 | 0.432 | 0.770 | 0.492 | 0.625 | 0.922 | 0.846 | 1.000 |
| OR | 0.812 ± 0.082 (0.717-0.983) | 0.852 ± 0.058 (0.773-0.975) | 0.846 ± 0.027 (0.791-0.880) | 0.857 ± 0.052 (0.788-0.962) | 0.851 ± 0.027 (0.797-0.899) | 0.020 | 0.193 | 0.020 | 0.105 | 0.922 | 0.160 | 1.000 | 0.770 | 0.193 | 0.846 |
| UR | 0.993 ± 0.013 (0.955-1.000) | 0.987 ± 0.011 (0.961-0.999) | 0.986 ± 0.006 (0.972-0.992) | 0.975 ± 0.024 (0.913-0.996) | 0.980 ± 0.013 (0.951-0.991) | 0.014 | 0.084 | 0.006 | 0.049 | 0.193 | 0.006 | 0.105 | 0.232 | 0.002 | 1.000 |
| GI | 1.447 ± 0.087 (1.315-1.624) | 1.470 ± 0.072 (1.349-1.587) | 1.449 ± 0.068 (1.357-1.573) | 1.494 ± 0.093 (1.386-1.699) | 1.491 ± 0.163 (1.329-1.884) | 0.160 | 0.557 | 0.131 | 0.695 | 0.492 | 0.492 | 0.770 | 0.027 | 1.000 | 0.193 |
| HI | 0.065 ± 0.011 (0.050-0.086) | 0.077 ± 0.013 (0.048-0.093) | 0.085 ± 0.008 (0.074-0.106) | 0.093 ± 0.023 (0.058-0.141) | 0.093 ± 0.016 (0.078-0.122) | 0.010 | 0.006 | **0.002** | **0.004** | 0.131 | **0.002** | 0.014 | 0.432 | 0.037 | 0.625 |
| Motor cortex |  |  |  |  |  |  |  |  |  |  |  |  |  |  |  |
| D_01%_ [Gy] | 61.097 ± 0.837 (59.82-62.40) | 61.280 ± 1.079 (59.54-63.30) | 61.645 ± 1.313 (58.92-63.17) | 61.039 ± 1.421 (58.58-62.76) | 61.574 ± 1.765 (57.26-63.10) | 0.846 | 0.102 | 0.695 | 0.125 | 0.201 | 0.492 | 0.391 | 0.020 | 0.846 | 0.275 |
| D_mean_ [Gy] | 54.302 ± 6.647 (42.671-60.817) | 48.201 ± 10.193 (32.038-57.673) | 46.789 ± 11.234 (30.704-57.652) | 48.086 ± 10.184 (32.162-57.859) | 47.080 ± 11.403 (30.901-58.056) | **0.002** | **0.002** | **0.002** | **0.002** | 0.020 | 0.557 | 0.049 | 0.014 | 0.049 | 0.105 |
| Motor cortex without PTV |  |  |  |  |  |  |  |  |  |  |  |  |  |  |  |
| D_01%_ [Gy] | 59.604 ± 1.750 (56.95-62.51) | 55.981 ± 2.317 (52.13-59.12) | 54.528 ± 2.351 (48.58-58.65) | 55.082 ± 2.351 (51.15-58.64) | 54.613 ± 2.294 (50.39-58.56) | **0.004** | **0.002** | **0.002** | **0.002** | 0.064 | 0.027 | 0.131 | 0.432 | 1.000 | 0.557 |
| D_mean_ [Gy] | 50.613 ± 7.339 (34.856-58.495) | 38.429 ± 9.007 (19.967-49.969) | 35.213 ± 8.700 (17.966-47.171) | 38.602 ± 9.035 (20.071-50.393) | 35.722 ± 8.574 (18.422-47.174) | **0.002** | **0.002** | **0.002** | **0.002** | **0.002** | 0.105 | **0.002** | **0.002** | 0.020 | **0.002** |
| Ipsilateral Hippocampus |  |  |  |  |  |  |  |  |  |  |  |  |  |  |  |
| D_01%_ [Gy] | 46.583 ± 18.054 (8.40-62.40) | 46.313 ± 17.969 (9.16-61.73) | 45.356 ± 19.662 (6.66-61.20) | 40.899 ± 19.621 (5.44-61.77) | 40.944 ± 20.885 (3.83-62.32) | 0.643 | 0.232 | **0.004** | 0.006 | 0.322 | **0.004** | 0.010 | 0.105 | 0.020 | 0.770 |
| D_mean_ [Gy] | 29.564 ± 18.300 (7.908-60.252) | 28.965 ± 17.285 (7.991-58.766) | 25.427 ± 20.190 (2.515-57.116) | 22.985 ± 18.233 (3.540-56.916) | 21.090 ± 18.203 (1.653-56.254) | 0.322 | 0.010 | **0.002** | **0.002** | 0.027 | **0.002** | **0.002** | 0.084 | **0.002** | **0.002** |
| Contralateral Hippocampus |  |  |  |  |  |  |  |  |  |  |  |  |  |  |  |
| D_01%_ [Gy] | 19.773 ± 12.593 (2.22-42.63) | 19.205 ± 11.437 (2.22-38.86) | 21.468 ± 11.131 (2.18-39.74) | 14.223 ± 10.350 (1.72-35.10) | 13.984 ± 10.456 (2.08-33.08) | 0.758 | 0.375 | **0.002** | 0.037 | 0.244 | **0.002** | 0.027 | **0.002** | **0.002** | 0.449 |
| D_mean_ [Gy] | 9.361 ± 7.907 (1.369-26.273) | 9.190 ± 7.598 (1.322-25.269) | 8.527 ± 6.033 (1.303-18.850) | 5.879 ± 4.807 (1.176-13.867) | 4.737 ± 3.655 (1.249-12.027) | 0.432 | 0.557 | **0.002** | **0.002** | 0.695 | **0.002** | **0.002** | **0.002** | **0.002** | 0.010 |
| Brain without PTV |  |  |  |  |  |  |  |  |  |  |  |  |  |  |  |
| D_01%_ [Gy] | 57.926 ± 1.41 (55.14-59.96) | 57.282 ± 1.164 (55.17-58.95) | 57.286 ± 0.326 (56.53-57.69) | 57.119 ± 1.229 (54.86-58.81) | 57.153 ± 0.347 (56.59-57.74) | 0.014 | 0.064 | 0.014 | 0.074 | 0.980 | 0.066 | 0.695 | 0.846 | 0.432 | 0.977 |
| D_mean_ [Gy] | 17.333 ± 3.425 (12.396-23.041) | 16.902 ± 3.169 (12.101-21.928) | 16.650 ± 2.709 (12.284-20.023) | 16.629 ± 2.892 (12.211-20.998) | 16.212 ± 2.576 (12.230-19.431) | **0.004** | 0.232 | **0.002** | 0.037 | 0.557 | 0.027 | 0.160 | 0.770 | **0.002** | 0.275 |
| Brainstem |  |  |  |  |  |  |  |  |  |  |  |  |  |  |  |
| D_01%_ [Gy] | 29.012 ± 18.271 (8.56-55.61) | 28.643 ± 17.502 (8.43-54.28) | 22.980 ± 18.954 (3.12-49.62) | 23.882 ± 18.770 (5.33-54.74) | 18.823 ± 17.968 (2.70-48.07) | 0.160 | **0.004** | **0.002** | **0.002** | **0.004** | **0.004** | **0.002** | 0.322 | 0.014 | **0.004** |
| D_mean_ [Gy] | 10.901 ± 7.059 (4.543-27.088) | 10.933 ± 6.905 (4.696-27.222) | 6.501 ± 6.959 (1.174-22.241) | 8.402 ± 6.430 (2.822-22.066) | 5.237 ± 6.085 (1.227-21.101) | 0.770 | 0.006 | **0.002** | **0.002** | 0.006 | **0.002** | **0.002** | 0.049 | 0.006 | **0.002** |
| Ipsilateral Thalamus |  |  |  |  |  |  |  |  |  |  |  |  |  |  |  |
| D_01%_ [Gy] | 57.542 ± 5.062 (46.67-62.45) | 57.539 ± 4.865 (48.94-62.10) | 57.551 ± 4.556 (49.44-63.19) | 56.981 ± 5.481 (46.11-62.01) | 56.759 ± 6.751 (42.83-62.11) | 0.846 | 0.625 | 0.264 | 0.941 | 0.846 | 0.232 | 0.922 | 0.719 | 0.695 | 1.000 |
| D_mean_ [Gy] | 43.771 ± 14.928 (17.573-60.793) | 43.215 ± 15.227 (18.111-60.264) | 42.678 ± 15.106 (17.049-59.470) | 42.508 ± 15.574 (15.281-60.149) | 40.327 ± 16.368 (16.451-59.646) | 0.557 | 0.105 | 0.049 | **0.002** | 0.375 | 0.084 | 0.014 | 0.625 | 0.010 | 0.064 |
| Contralateral Thalamus |  |  |  |  |  |  |  |  |  |  |  |  |  |  |  |
| D_01%_ [Gy] | 35.717 ± 15.886 (14.56-59.92) | 36.111 ± 15.750 (11.31-60.03) | 37.720 ± 12.976 (17.28-58.20) | 34.398 ± 15.635 (12.40-59.64) | 34.633 ± 13.146 (15.10-57.89) | 0.695 | 0.131 | 0.055 | 0.492 | 0.275 | 0.014 | 0.375 | 0.027 | **0.004** | 1.000 |
| D_mean_ [Gy] | 24.505 ± 12.255 (8.805-45.872) | 24.552 ± 11.843 (8.992-45.003) | 26.293 ± 10.355 (9.393-38.941) | 22.058 ± 12.006 (6.959-41.956) | 22.353 ± 10.038 (8.618-38.501) | 0.770 | 0.375 | **0.002** | 0.131 | 0.232 | **0.004** | 0.160 | 0.014 | **0.004** | 0.922 |
| Medulla |  |  |  |  |  |  |  |  |  |  |  |  |  |  |  |
| D_01%_ [Gy] | 7.380 ± 1.271 (5.33-9.16) | 7.509 ± 1.266 (5.26-9.55) | 2.220 ± 2.042 (0.61-7.46) | 6.131 ± 2.470 (2.38-9.67) | 2.517 ± 2.838 (0.73-10.27) | 0.574 | **0.002** | 0.064 | **0.004** | **0.002** | 0.027 | **0.004** | **0.002** | 0.652 | **0.004** |
| D_mean_ [Gy] | 5.109 ± 1.405 (2.265-6.985) | 5.208 ± 1.319 (2.703-6.796) | 1.325 ± 1.018 (0.317-3.773) | 3.962 ± 1.542 (1.527-6.900) | 1.545 ± 1.614 (0.498-5.931) | 0.492 | **0.002** | 0.027 | **0.002** | **0.002** | 0.049 | **0.002** | **0.002** | 0.625 | **0.004** |
| Ipsilateral Opticus nerve |  |  |  |  |  |  |  |  |  |  |  |  |  |  |  |
| D_01%_ [Gy] | 12.919 ± 15.547 (1.38-51.70) | 12.934 ± 15.517 (1.30-51.49) | 7.216 ± 12.625 (1.34-42.62) | 12.167 ± 16.103 (1.33-53.03) | 7.261 ± 11.751 (1.28-39.35) | 1.000 | **0.002** | 0.039 | **0.002** | **0.004** | 0.275 | **0.002** | **0.004** | 0.748 | **0.002** |
| D_mean_ [Gy] | 7.761 ± 10.622 (0.804-33.776) | 7.563 ± 10.003 (0.744-31.679) | 4.392 ± 6.802 (0.754-23.199) | 7.338 ± 10.542 (0.774-33.610) | 4.559 ± 6.556 (0.728-22.180) | 0.375 | **0.002** | **0.002** | **0.002** | **0.004** | 0.232 | **0.002** | **0.002** | 0.846 | **0.002** |
| Contralateral Opticus nerve |  |  |  |  |  |  |  |  |  |  |  |  |  |  |  |
| D_01%_ [Gy] | 8.229 ± 9.499 (1.28-33.41) | 7.547 ± 8.326 (1.25-29.03) | 2.899 ± 2.357 (1.26-9.26) | 7.724 ± 9.928 (1.21-34.34) | 3.008 ± 2.645 (1.23-10.07) | 0.020 | **0.002** | 0.100 | **0.002** | **0.004** | 0.695 | **0.002** | **0.004** | 0.898 | **0.004** |
| D_mean_ [Gy] | 4.421 ± 4.704 (0.816-15.894) | 4.044 ± 3.973 (0.808-13.100) | 1.768 ± 1.168 (0.800-4.696) | 4.605 ± 5.615 (0.784-19.184) | 1.809 ± 1.265 (0.786-4.915) | 0.014 | **0.002** | 0.557 | **0.002** | **0.002** | 0.846 | **0.004** | **0.004** | 0.557 | **0.004** |
| Ipsilateral lens |  |  |  |  |  |  |  |  |  |  |  |  |  |  |  |
| D_01%_ [Gy] | 2.299 ± 1.380 (0.29-4.30) | 2.166 ± 1.270 (0.26-4.24) | 1.709 ± 1.285 (0.27-4.54) | 2.209 ± 1.238 (0.28-4.30) | 1.758 ± 1.345 (0.24-4.62) | 0.012 | 0.014 | 0.680 | 0.061 | 0.045 | 0.289 | 0.064 | 0.020 | 0.482 | 0.064 |
| D_mean_ [Gy] | 1.926 ± 1.170 (0.237-3.950) | 1.780 ± 1.074 (0.215-3.808) | 1.454 ± 1.127 (0.215-4.034) | 1.818 ± 1.086 (0.225-3.907) | 1.519 ± 1.213 (0.187-4.117) | **0.002** | 0.010 | 0.105 | 0.049 | 0.105 | 0.131 | 0.131 | 0.037 | 0.375 | 0.105 |
| Contralateral lens |  |  |  |  |  |  |  |  |  |  |  |  |  |  |  |
| D_01%_ [Gy] | 2.187 ± 1.349 (0.53-3.89) | 2.073 ± 1.249 (0.41-3.33) | 1.257 ± 0.752 (0.44-2.60) | 2.178 ± 1.256 (0.41-3.87) | 1.397 ± 0.833 (0.45-2.72) | 0.336 | **0.004** | 0.938 | **0.004** | 0.008 | 0.313 | 0.008 | 0.008 | 0.027 | 0.008 |
| D_mean_ [Gy] | 1.891 ± 1.188 (0.451-3.534) | 1.739 ± 103.1 (0.374-2.855) | 1.090 ± 0.662 (0.397-2.439) | 1.814 ± 1.056 (0.372-3.309) | 1.188 ± 0.706 (0.414-2.540) | 0.055 | **0.004** | 0.164 | **0.004** | 0.008 | 0.203 | 0.008 | 0.008 | 0.027 | 0.008 |
| Chiasma |  |  |  |  |  |  |  |  |  |  |  |  |  |  |  |
| D_01%_ [Gy] | 18.341 ± 15.014 (3.67-53.88) | 18.447 ± 14.846 (3.78-53.70) | 9.288 ± 13.819 (2.52-47.75) | 16.477 ± 15.647 (3.52-54.00) | 9.277 ± 13.716 (2.32-47.50) | 0.922 | **0.002** | 0.006 | **0.002** | **0.002** | 0.049 | **0.002** | **0.002** | 0.980 | **0.002** |
| D_mean_ [Gy] | 12.989 ± 13.674 (2.100-48.346) | 12.808 ± 13.756 (2.063-48.459) | 6.875 ± 11.557 (1.813-39.505) | 11.816 ± 14.104 (1.952-48.610) | 6.949 ± 11.362 (1.798-38.890) | 0.375 | **0.002** | 0.006 | **0.002** | **0.002** | 0.160 | **0.002** | **0.002** | 0.846 | **0.002** |
| Ipsilateral cochlea |  |  |  |  |  |  |  |  |  |  |  |  |  |  |  |
| D_01%_ [Gy] | 10.179 ± 7.410 (5.86-30.58) | 9.826 ± 7.641 (3.77-30.61) | 5.168 ± 9.376 (0.76-31.66) | 7.963 ± 8.477 (1.67-30.41) | 5.083 ± 9.277 (0.87-31.28) | 0.820 | **0.004** | **0.002** | **0.004** | **0.004** | 0.010 | **0.004** | 0.014 | 0.559 | 0.008 |
| D_mean_ [Gy] | 8.275 ± 4.676 (4.187-20.147) | 8.058 ± 4.921 (2.398-20.088) | 3.669 ± 5.590 (0.667-19.325) | 6.353 ± 5.513 (1.389-19.689) | 3.659 ± 5.673 (0.683-19.537) | 0.770 | **0.002** | **0.002** | **0.002** | **0.002** | 0.006 | **0.002** | **0.004** | 0.770 | **0.002** |
| Contralateral cochlea |  |  |  |  |  |  |  |  |  |  |  |  |  |  |  |
| D_01%_ [Gy] | 2.546 ± 3.745 (0.80-13.12) | 2.540 ± 3.819 (0.77-13.34) | 1.435 ± 0.830 (0.63-3.52) | 2.092 ± 2.957 (0.64-10.42) | 1.277 ± 0.789 (0.59-3.28) | 0.531 | 0.012 | **0.002** | **0.002** | 0.025 | **0.002** | **0.002** | 0.822 | **0.002** | **0.002** |
